# Nanomedicines for Dry Eye Syndrome: Targeting Oxidative Stress with Modern Nanomaterial Strategies

**DOI:** 10.3390/molecules29163732

**Published:** 2024-08-07

**Authors:** Aleksandra Krawczyk, Sara Marta Stadler, Barbara Strzalka-Mrozik

**Affiliations:** Department of Molecular Biology, Faculty of Pharmaceutical Sciences in Sosnowiec, Medical University of Silesia, 40-055 Katowice, Poland; s81894@365.sum.edu.pl (A.K.); s81915@365.sum.edu.pl (S.M.S.)

**Keywords:** dry eye syndrome, oxidative stress, nanomaterials, antioxidants, drug delivery system

## Abstract

Dry eye syndrome (DES) is a dynamic, chronic disease of the ocular surface and ocular appendages caused by inflammation. The most common symptoms include redness, itching, and blurred vision, resulting from dysfunction of the meibomian glands and impaired tear-film production. Factors contributing to the development of DES include environmental elements, such as UV radiation, and internal elements, such as hormonal imbalances. These factors increase oxidative stress, which exacerbates inflammation on the surface of the eye and accelerates the development of DES. In recent years, the incidence of DES has risen, leading to a greater need to develop effective treatments. Current treatments for dry eye are limited and primarily focus on alleviating individual symptoms, such as reducing inflammation of the ocular surface. However, it is crucial to understand the pathomechanism of the disease and tailor treatment to address the underlying causes to achieve the best possible therapeutic outcomes. Therefore, in this review, we analyzed the impact of oxidative stress on the development of DES to gain a better understanding of its pathomechanism and examined recently developed nanosystems that allow drugs to be delivered directly to the disease site.

## 1. Introduction

Dry eye syndrome (DES) is a chronic, multifactorial disease that affects the surface of the eye, characterized by progressive inflammation leading to inadequate or abnormal tear-film production [1,2]. It is among the most prevalent conditions affecting the ocular surface, particularly prevalent in individuals aged over 50 years and more frequently observed in women [3]. Factors contributing to its prevalence include compromised immunity and various underlying systemic, hereditary, and autoimmune diseases, as well as the use of multiple medications concurrently [1,4,5].

Corneal neurosensory abnormalities are a prominent feature of dry eye syndrome, arising from disturbances in corneal innervation. The cornea is the most densely innervated tissue in the human body, facilitating rapid transmission of sensory information to higher brain centers [6]. Common symptoms associated with these abnormalities include eye redness, itching, a burning sensation, blurred vision, sensitivity to light, and the feeling of a foreign body in the eye [6]. In severe cases of DES, vision loss can also occur [1,6].

Current treatments for diseases affecting the anterior segment of the eye rely heavily on eye drops. Despite the availability of potent medications, many of them fail to achieve the desired therapeutic outcomes due to limited bioavailability. Moreover, frequent dosing requirements contribute to increased treatment costs. Although intraocular injections offer improved bioavailability, their high patient burden limits their current use. Consequently, efforts have been focused on developing novel drug delivery systems that enhance bioavailability and are non-toxic. These systems utilize nanomaterials, in situ forming gels, and combinations thereof [7,8,9].

Nanomaterials are characterized by their small size and dynamic properties, which confer high bioavailability due to their physicochemical features such as shape, size, and structure. There are various types of nanomaterials including polymers, carbohydrates, and metallic and non-metallic nanomaterials. By modifying their synthesis methods, it is possible to control these properties to enhance their biological effects [10,11,12].

In this review, we will initially focus on the most common causes and pathophysiology of dry eye syndrome, highlighting the role of oxidative stress in its development. Subsequently, we will discuss state-of-the-art treatments aimed at reducing oxidative stress, with a specific emphasis on the use of nanomaterials.

## 2. Common Causes and Pathophysiology of Dry Eye Syndrome

There are numerous factors contributing to the development of DES, categorized into environmental (external stimuli) and internal (internal physiological factors) factors (Table 1). Key environmental factors include UV radiation, dry and windy climates, and air pollution [1,4,5]. Internal factors encompass hormonal imbalances, autoimmune conditions, nerve damage, and gastrointestinal dysfunction. Each of these conditions plays a role in the pathogenesis of dry eye syndrome [13,14].

The eye surface is a complex structure composed of multiple tissues that interact with the circulatory, nervous, and hormonal systems to maintain proper eye function, including tear-film secretion [25]. The primary causes of DES are deficiency in the lipid layer and dysfunction of the meibomian glands [26].

Meibomian glands are sebaceous glands located in the eyelids; they secrete meibum, which reduces surface tension and stabilizes the tear film. The most common dysfunction of the meibomian glands involves obstruction of their openings and reduced efficiency in delivering the oily component of the tear film, namely meibum. This dysfunction leads to accelerated tear evaporation, diminished expansion of the tear film, inflammation, and an increase in reactive oxygen species (ROS) levels, all of which exacerbate dry eye syndrome. Obstructed meibomian glands may also contribute to the development of micro-injuries on the eye’s surface due to frequent blinking [2,27].

**Figure 1:**
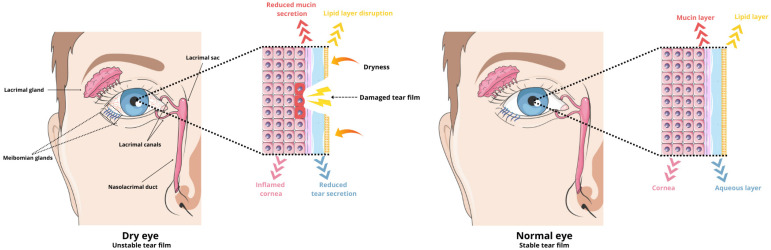
Schematic of dysregulated tear film during DES and normal tear film.

The tear film is considered the antioxidant defense system in the anterior segment of the eye. It is composed of three layers: a lipid layer (outer layer), a water layer (middle layer), and a mucous layer (inner layer) (Table 2) [28]. This film contains various antioxidant enzymes, such as superoxide dismutase and glutathione peroxidase, which are crucial for maintaining the homeostasis of the ocular surface. It acts as a protective barrier between the external and internal environments of the eye, guarding against infections and mechanical damage while providing nourishment and oxygenation to the eye structures. However, factors such as aging, certain medications, or a poor diet can disrupt the function and composition of the tear film (Figure 1). This disruption leads to inflammation and the production of hyperosmolar tears [29,30,31,32].

Micro-injuries to the corneal epithelium have significant implications for the adhesion of mucins to the eye surface. Mucins, glycoproteins responsible for lubrication and minimizing friction during blinking, play a crucial role. Without adequate mucins, the cornea becomes hydrophobic, impairing the ability of the tear film’s aqueous components to function effectively. This leads to increased tear evaporation and destabilization of the tear film, triggering immune system activation and the onset of chronic inflammation [1,33].

Disturbances in the tear film, dysfunction of the meibomian glands, environmental factors, and internal factors are among the numerous causes contributing to the development of dry eye syndrome. It is essential to note that the treatment approach for DES varies based on the specific symptoms and underlying pathophysiology [28].

## 3. Oxidative Stress

Oxidative stress has been extensively studied for centuries, highlighting its profound connection with disruptions in oxidation–reduction (redox) homeostasis. This imbalance triggers redox reactions integral to nearly all metabolic processes, resulting in the overproduction of free radicals. These radicals encompass reactive nitrogen species (RNS), reactive oxygen species (ROS), reactive sulfur species (RSS), reactive carbonyl species (RCS), and reactive selenium species (RSeS). Among these, RNS and ROS are particularly significant, as their excess inhibits antioxidant mechanisms crucial for maintaining bodily equilibrium. Consequently, oxidative stress is implicated in the development of various disorders, including neurodegenerative diseases, degenerative conditions, cancers, and ocular diseases [37,38,39].

### 3.1. Oxidative Stress Classification

Due to the wide array of compounds involved in oxidative stress pathogenesis, its complexity, and the lack of a reliable technique for assessing ROS levels, there is currently no universally accepted classification of oxidative stress. Efforts are ongoing to develop a standardized method for assessing ROS levels and establishing a classification framework. Initial steps have been taken toward this goal, proposing classifications based on intensity and time course. Intensity-based classification aims to quantify the concentration of ROS-modified molecules and the activity of antioxidant enzymes, allowing differentiation between toxic oxidative stress and physiological levels (Table 3). However, experimental validation of this classification framework is still lacking [38,40].

Classification based on the time course considers the duration of persistently elevated ROS levels triggered by factors such as oxidative stress inducers, accompanied by alterations in gene expression aimed at neutralizing oxidative stress (Table 4). Chronic oxidative stress is categorized into at least two subtypes: sustained ROS levels persistently higher than baseline or intermittent ROS levels occasionally exceeding the normal range [38,40].

### 3.2. Role of Oxidative Stress in the Development of Dry Eye Disease

Under physiological conditions, free radicals are produced in moderate amounts to support immune functions and participate in cellular respiration [41]. However, when produced in excess, they can cause significant cellular damage.

Cell membranes are particularly susceptible to damage due to their high lipid content. Reactive oxygen species react with iron, initiating lipid peroxidation. This process disrupts the integrity of cell membranes, interferes with signaling pathways, triggers inflammatory responses, and can even lead to apoptosis [42,43].

Additionally, ROS induce various types of oxidative damage to DNA and RNA, altering gene expression and disrupting intercellular information transfer, thereby compromising organ and tissue functionality. DNA dysfunction can also impact protein expression and modification, impairing numerous enzymatic reactions and signal transduction pathways [41,44].

The eye, positioned and structured as it is, is highly vulnerable to cellular damage due to the compounded impact of external reactive oxygen species on its anterior segment and diminished antioxidant defenses. External sources of ROS encompass direct exposure to oxygen, light, and ultraviolet radiation. These factors disrupt oxidation processes, leading to molecular alterations in ocular structures and consequently contributing to the onset of oxidative stress-related eye conditions such as dry eye syndrome [37].

## 4. Nanomaterials and Drug Delivery to the Eye

Nanocarriers are composed of particles ranging in size from 10 to 1000 nm and possess specific surface charges. These diverse sizes enable numerous applications in the biomedical field. The surface charge plays a crucial role in retaining nanocarriers at targeted locations. Zeta potential (ZP) serves as an indicator of the physical stability of these nanosystems. A ZP value around ±20 mV is optimal for electrostatic attachment to the corneal surface This parameter is crucial for maintaining the stability of nanodispersions. When particles possess high Zeta potential with the same charge, they repel each other due to repulsive forces, which prevents their aggregation [45].

In ophthalmic delivery, the cornea and conjunctiva surfaces typically carry a negative charge. Therefore, cationic nanoparticles can be attracted to these surfaces through electrostatic interactions. This attraction facilitates the adherence and retention of nanoparticles on the ocular tissues, improving drug-delivery efficiency [45].

The retention of cationic nanoparticles on negatively charged eye tissues facilitates localized drug delivery to the anterior segment of the eye. In contrast, when cationic nanoparticles are injected intravitreally, they disperse and accumulate throughout the vitreous, while anionic particles have the capability to diffuse into the retina. The ability of nanocarriers to deliver therapeutics to specific sites within the eye is attributed to their nanoscale size and surface properties [45].

Nanomedicines encompass polymer–drug conjugates and nanoparticle systems, which share similarities due to the extensive advancements in drug delivery technologies [45].

According to World Health Organization statistics, at least 2.2 billion people suffer from visual impairment or blindness, with approximately half of these cases being preventable [46]. This statistic highlights the critical need for advanced drug delivery strategies in ocular therapies to address and potentially reduce preventable visual impairments.

Traditionally, drugs are delivered to the eye through local or systemic routes. These include a variety of delivery systems such as eye drops (solutions, suspensions, and emulsions), in situ gelling formulations, eye pads, contact lenses, punctal plugs, intraocular injections, and implants, all designed to enhance the effectiveness of drug delivery to the eyes [47].

**Figure 2:**
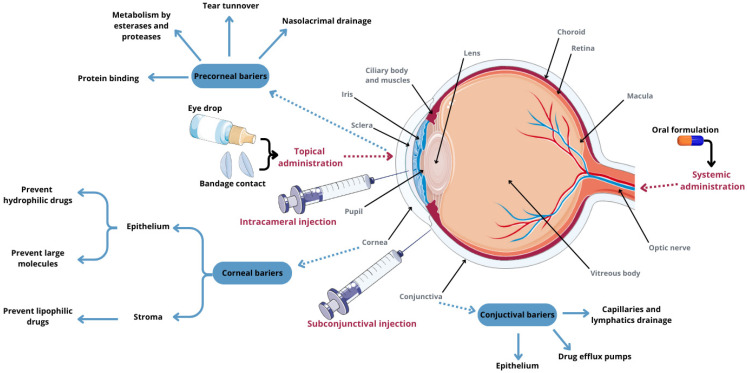
Eye anatomy, routes of administration for anterior-segment drug delivery, and ocular tissue barriers that prevent drug absorption into the eye. Parts of the figure were drawn using pictures from Servier Medical Art, licensed under Creative Commons Attribution 4.0 International.

Conventional topical drug administration plays a crucial role in treating eye diseases, but it suffers from low drug availability. Less than 5% of the total dose administered via eye drops reaches the inner tissues of the eye. The small surface area of the eye limits the volume of liquid formulation that can be applied to about 30 μL. Moreover, most of the drug is quickly removed from the ocular surface due to lacrimal turnover, blinking, and nasolacrimal drainage (Figure 2) [47]. This necessitates frequent instillation of eye drops throughout the day, which can cause inflammation of the ocular surface and temporary blurred vision. Moreover, long-term frequent use of topical medications can lead to discomfort and damage to the ocular surface. Conversely, invasive intraocular injection surgery increases patient reluctance due to potential side effects and complications [48,49].

Conventional ophthalmic therapy faces numerous anatomical barriers, making it challenging to effectively deliver drugs to the ocular surface. Recent advancements in bioadhesive gelation systems in situ and nanotechnology-based drug delivery systems are increasingly sought after to overcome these challenges. Nanocarrier-based therapeutic delivery systems have been developed to facilitate sustained and targeted drug delivery to both the anterior and posterior segments of the eye, thereby reducing side effects [48,49].

**Figure 3:**
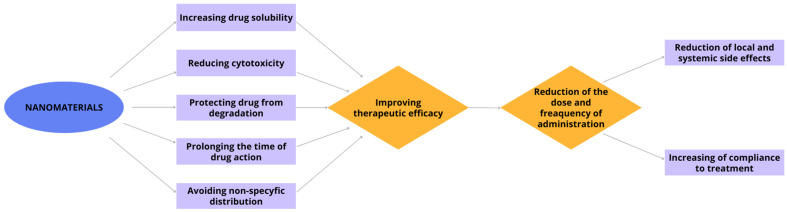
Advantages of the use of nanomaterials in ophthalmic diseases.

The utilization of nanomaterials for drug delivery to the eye enhances the efficacy of therapies for eye diseases (Figure 3) [47,49].

### 4.1. Polymer Nanomaterials

Polymeric nanomaterials encompass various types such as polymeric nanoparticles (NPs), polymeric micelles, dendrimers, polymer hydrogels, and polymer nanofibers. These materials can be engineered with specific properties tailored to their intended applications [50].

#### 4.1.1. Polymeric Nanoparticles

Polymer nanoparticles are categorized into nanospheres, which are solid spheres formed from cross-linked polymers and nanocapsules, which have a small liquid core surrounded by a polymer membrane. In nanospheres, the drug is either adsorbed on the surface or trapped within the particle, whereas in nanocapsules, the drug can be adsorbed on the surface of the capsule or encapsulated within the liquid core [51]. These nanoparticles are composed of synthetic polymers such as polylactic acid, poly-L-lysine, polyglycolic acid, and polyethylene glycol or natural polymers like gelatin, chitosan, heparin, starch, and CS [50,51]. They are known for their enhanced bioavailability, adhesion, and prolonged residence time [49].

##### Poly(Lactic and Co-Glycolic Acid) Nanoparticles (PLGA NP) Encapsulating Xanthohumol

PLGA nanoparticles (NPs) serve as safe carriers for delivering hydrophobic molecules to the ocular surface. Ghosh et al. [52] demonstrated that PLGA NPs encapsulating xanthohumol, a natural compound found in hops known for enhancing the endogenous antioxidant response and directly neutralizing reactive oxygen species (ROS) as a polyphenol chalcone, reduced oxidative DNA damage in corneal epithelial cells in vivo in a mouse model of dry eye syndrome induced by drying stress and scopolamine. This study confirmed the cytoprotective effect of xanthohumol-encapsulating PLGA NPs against oxidative stress-induced damage in human corneal epithelial cells (HCE-T) in vitro [52].

**Figure 4:**
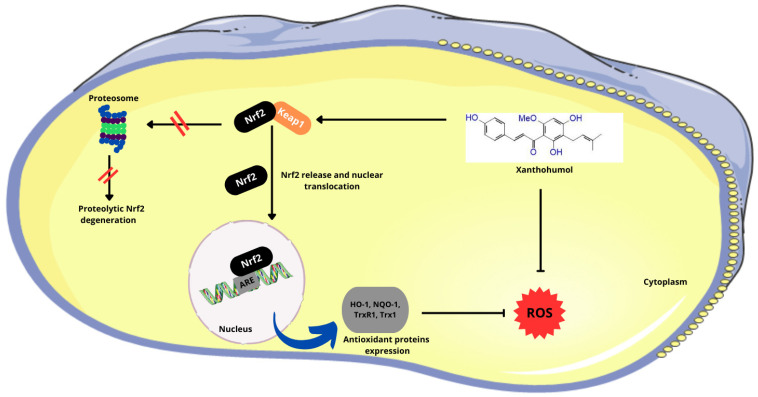
Effect of the α,β-unsaturated ketone structure in Xn on the Nrf2 pathway. (Nrf2—nuclear factor erythroid 2-related factor 2; Keap1—Kelch-like ECH-associated protein 1; ARE—antioxidant-responsive element; HO-1—heme oxygenase 1; NQO1—NAD(P)H:quinone oxidoreductase 1; Trx1—thioredoxin 1; TrxR1—thioredoxin reductase 1; ROS—reactive oxygen species). Parts of the figure were drawn using pictures from Servier Medical Art, licensed under Creative Commons Attribution 4.0 International.

After PLGA NPs release xanthohumol into the cell, the α,β-unsaturated ketone structure of xanthohumol allows it to covalently bind to a cysteine residue in the cytosolic repressor protein Kelch-like ECH-associated protein 1 (Keap1). This binding prevents Keap1 from targeting the nuclear factor erythroid 2-related factor 2 (Nrf2) for degradation via the proteasome pathway. As a result, Nrf2 is stabilized and translocates to the nucleus, where it binds to antioxidant-responsive element (ARE) sequences in the genome. This binding activates the transcription of antioxidant genes, thereby enhancing the cellular antioxidant response (Figure 4) [53].

##### Poly(Catechin) Capped-Gold Nanoparticles (Au@Poly-CHNPs) Carrying Amfenac [AF; A Nonsteroidal Anti-Inflammatory Drug (NSAID)]

**Figure 5:**
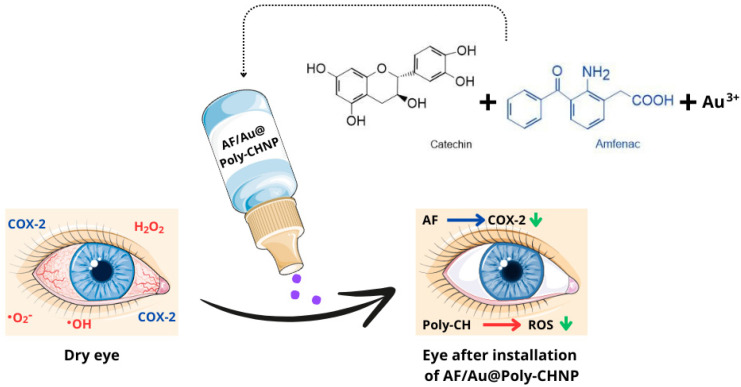
Diagram of AF/Au@Poly-CH NPs administration. (COX-2—cyclooxygenase 2; •O_2_^−^—superoxide anion radical; •OH—hydroxyl radical; H_2_O_2_—hydrogen peroxide; AF—amphenac; Poly-CH—Poly-catechin; ROS—reactive oxygen species). Parts of the figure were drawn using pictures from Servier Medical Art, licensed under Creative Commons Attribution 4.0 International.

Au@Poly-CH NPs are composed of catechin (CH), gold nanoparticles, and amfenac (AF). Gold nanoparticles are synthesized through a redox reaction between tetrachloroauric (III) acid (HAuCl4) and catechin, where gold forms the core and catechin forms the shell [54]. Amfenac (2-amino-3-benzobenzeneacetic acid) is a nonsteroidal anti-inflammatory drug (NSAID) and the active metabolite of nepafenac. AF effectively inhibits the activity of both cyclooxygenase-1 (COX-1) and cyclooxygenase-2 (COX-2) enzymes [55]. This novel treatment developed by Li et al. [56] aims to mitigate damage to ocular surface tissue in dry eye syndrome by concurrently addressing cyclooxygenase-induced inflammation and ROS-induced oxidative stress (Figure 5). Researchers have demonstrated that Au@Poly-CH nanoparticles exhibit notable activity in scavenging superoxide anions [56].

Antioxidant and anti-inflammatory effects of these nanoparticles were tested on a rabbit model of dry eye syndrome and assessed using DCFH-DA for oxidative stress and prostaglandin E2/VEGF assays for inflammation, respectively. Hematoxylin and eosin (H&E) staining revealed improvements in corneal normality and thickness compared to the control group, with a significant increase in the number of goblet cells. These findings suggest that Au@PolyCH nanoparticles loaded with nonsteroidal anti-inflammatory drugs represent a promising multifunctional nanocomposite for treating DES [56,57].

##### PLGA Nanoparticles Loaded with Cyclosporine A (CsA) and Cyclosporine A Lipid Nanocapsules (CsA-LNC)

**Figure 6:**
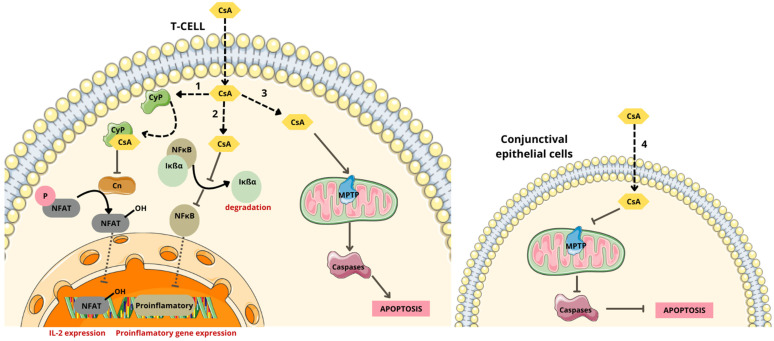
Mechanism of action of cyclosporine A. (1) Binds with cyclophilin (CyP) complex and inhibits dephosphorylation of nuclear factor of activated T (NFAT) cells, by inhibiting calcineurin (Cn) activation and the subsequent release of interleukin 2. (2) Inhibits nuclear factor κB (NFκB) activation by inhibiting the phosphorylation, ubiquitination, and subsequent degradation by the proteosome of inhibitor-of-kappaB (IκBα) bound to NF-κB and the subsequent release of pro-inflammatory cytokines. (3) Induces T cell apoptosis, caspase activation, and mitochondrial permeability transition pore (MPTP) opening. (4) Inhibits intrinsic mitochondrial pathway, caspase activation, and apoptosis [61,65]. Parts of the figure were drawn using pictures from Servier Medical Art, licensed under Creative Commons Attribution 4.0 International.

Topical 0.05% cyclosporine A (CsA), formulated as a mixture of immiscible components and surfactants including castor oil, glycerin, polysorbate 80, is the first artificial therapeutic tear approved by the Food and Drug Administration (FDA) for the treatment of DES. Currently, CsA eye drops are primarily formulated in oily preparations [58,59]. Cyclosporine A, a potent immunosuppressive drug, exerts several effects including the prevention of opening transition pores of mitochondrial permeability, thereby inhibiting the release of cytochrome *c* and reducing apoptosis (Figure 6) [60,61]. CsA also plays a role in inhibiting apoptosis of conjunctival goblet cells and lacrimal lobular cells by regulating mucin synthesis and secretion [59]. Furthermore, the mechanism of action of cyclosporine A involves the inhibition of calcineurin activation [61].

Calcineurin translocates into the nucleus where it binds to the nuclear factor of activated T cells (NFAT), thereby modulating immune responses and inflammation. This complex is responsible for the transcription of pro-inflammatory cytokines in the nucleus of the cell, such as interleukins (IL-2, IL-3, IL-4, IL-5), tumor necrosis factor alpha (TNF-α), and transforming growth factor beta (TGF-β). These cytokines play crucial roles in immune responses, inflammation, and tissue remodeling processes [61].

CsA enters the cytoplasm of lymphocytes and binds to cyclophilin (CyP). This complex inhibits calcineurin (Cn), preventing the transcription of cytokine genes, particularly IL-2, which is crucial for T cell replication. Therefore, cyclosporine A is a potent inhibitor of T cell proliferation [61]. Additionally, CsA blocks the expression of immune mediators such as IL-1β, TNF-α, and especially IL-6, thereby inhibiting the recruitment of T lymphocytes and their immune response [62].

Studies have demonstrated that nanoparticles offer sustained drug release at the injection site and within ocular tissues, persisting for an extended period after uptake by epithelial cells. This interaction ensures optimal contact of the drug preparation with the ocular mucous membrane. CsA-loaded PLGA nanoparticles have shown significant potential as effective drug delivery systems [63].

The lipid nanoconstruct (LNC) consists of a lipid core surrounded by a cohesive coating with a tensile effect, serving as a versatile carrier for the nanoencapsulation or nano-association of drugs. Studies have demonstrated that CsA-LNCs effectively inhibit IL-2 production, thereby suppressing T lymphocyte activation. CsA-LNCs also promote corneal epithelial regeneration and exhibit anti-inflammatory effects in the cornea. Topical administration of CsA-LNCs has been shown to be more effective in treating dry eye syndrome (DES) compared to CsA emulsion, attributed to the higher bioavailability of CsA-LNCs in DES target tissues than CsA emulsion [64].

##### Polyglycolic Acid-Loaded Tetrandrine Nanoparticles

Tetrandrine (TET) is a bisbenzylisoquinoline alkaloid extracted from the Chinese plant *Stephania tetrandra* S.Moore [66]. It possesses various pharmacological effects, including anti-inflammatory properties, promotion of cell apoptosis, antiaggregation effects, blocking of Ca^2+^ channels, immunosuppressive effects, and scavenging of free radicals. However, due to its low solubility in water, TET has limited bioavailability in the eye and short retention time in the cornea [67,68].

Commercial formulations of TET, when administered topically, increase tear volume, leading to rapid drainage of excess fluid through the nasolacrimal duct, resulting in more than 85% of the administered dose being lost before reaching the corneal surface. Therefore, there is a critical need to develop an effective ocular delivery system that enhances corneal targeting and increases drug concentration on the corneal surface, thereby improving therapeutic efficacy [68].

Polyglycolic acid-loaded tetrandrine nanoparticles (Tet-ATS@PLGA) in a rabbit model of dry eye syndrome were examined. Artificial tear substitutes (ATSs) were also employed to lubricate the cornea and reduce tear evaporation [69]. Studies demonstrated that Tet-ATS@PLGA nanoparticles had a Zeta potential of 23.58 ± 0.78 mV, which enhances their adaptation to the negatively charged environment of the ocular surface following modification. Furthermore, this nanodrug effectively induced apoptosis in inflammatory corneal epithelial cells, leading to inhibition of the expression of vascular endothelial growth factor (VEGF), interleukin-1β (IL-1β), prostaglandin E2 (PGE2), and tumor necrosis factor-α (TNF-α). This positive effect contributed to the recovery of corneal epithelial thickness. Although the lacrimal gland in DES rabbits did not fully recover after 2 weeks of treatment (with the amount of tears secreted still lower than in healthy rabbits), there was significant improvement observed. Additionally, tetrandrine also contributed to reducing intraocular pressure [68].

In conclusion, Tet-ATS@PLGA nanoparticles represent a promising new approach for the treatment of dry eye syndrome.

##### Grape Seed Nanochats

**Figure 7:**
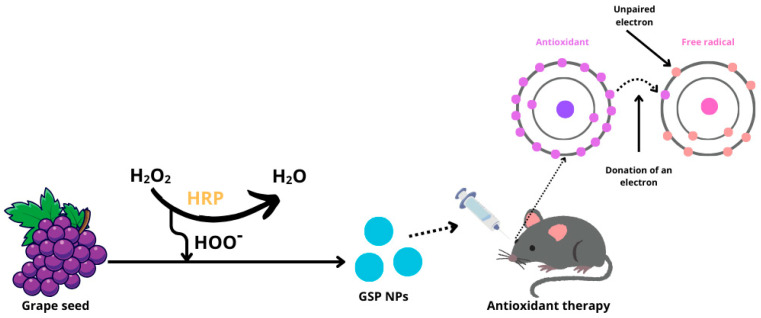
Production of GSP nanoparticles and the effect of antioxidants on free radicals. (H_2_O_2_—hydrogen peroxide; HRP—horseradish peroxidase; HOO^−^—hydroperoxyl radical; GSP NPs—grape seed nanoparticles).

The study published by Wang et al. [70] introduces GSP nanoparticles (GSP NPs) synthesized from grape seeds using polymerization in the presence of the enzyme horseradish peroxidase (HRP) and hydrogen peroxide. They investigated the antioxidant effects of these nanoparticles on DES using an experimental mouse model where benzalkonium chloride was locally applied to induce tear-film cracking and drying (Figure 7).

Grape seeds are a concentrated source of polyphenolic compounds, including procyanidin, catechin, epicatechin, gallocatecol, and gallic acid, noted for their excellent biocompatibility and bioavailability [71]. Polyphenols act as potent antioxidants, effectively reducing the production of free radicals through the Fenton reaction [72]. Their structure allows for the delocalization of electrons, enabling them to capture radicals and inhibit the initiation stage of chain oxidation, thus preventing lipid oxidation [73]. Additionally, polyphenols can act as radical scavengers by transferring a hydrogen atom from their active hydroxyl groups to free radicals, resulting in the formation of a phenolic radical and a stable quinone structure. These phenolic radicals can further react with other free radicals to enhance their antioxidant effect [74]. It has been demonstrated that eye drops containing grape seed polyphenol nanoparticles effectively control oxidative stress levels and reduce apoptosis rates in the corneal epithelial and conjunctival cells of mice with dry eye syndrome [70].

##### Gelatin-Based Nanoparticles

Gelatin is a natural polymer derived from collagen and has gained significant attention in recent years due to its favorable properties, including its structure, non-toxicity, biodegradability, and multifunctionality. Gelatin is produced through the partial hydrolysis of collagen using bases, heat, and acids. It contains the amino acid sequence arginine-glycine-aspartic acid, which facilitates modification of cell adhesion. Given its structural characteristics and the abundance of functional groups, gelatin’s structure and function can be modified by adjusting factors such as temperature, gelatin concentration, and the energy required to form secondary structures. This versatility enables gelatin to be used for targeted drug delivery to cells and tissues. By varying the cross-linking density of gelatin particles, different drug release profiles can be achieved, allowing for personalized treatment tailored to the severity of dry eye syndrome [75,76,77].

Several techniques have been developed to produce gelatin-based nanomaterials for drug delivery systems (DDSs), including mechanical methods (such as emulsion and spray drying) and physicochemical methods (such as precipitation and desolvation). Regardless of the technique used, a common step involves the use of chemical cross-linking agents (such as formaldehyde, glutaraldehyde, and genipin) to achieve the desired degradation profiles for DDSs [78,79].

In the study, gelatin was combined with cinnamic acid to create a photocross-linked hydrogel (GelCA). Cinnamic acid contains carboxyl groups that facilitate chemical cross-linking with gelatin lysine residues through amide bonds. Additionally, the phenyl group in cinnamic acid enhances the affinity of GelCA for the adsorption of lipophilic molecules, thereby supporting the development of effective drug delivery systems [75,80,81].

It has been demonstrated that GelCA alters its viscosity when exposed to UV radiation, transforming into a slightly elastic material that is resistant to stretching and mechanical damage. The study utilized three types of GelCA hydrogels: GelCA alone, GelCA combined with polydopamine (PDA@GelCA) nanoparticles, and GelCA combined with curcumin-loaded polydopamine nanoparticles (Cur@PDA@GelCA) [82]. Cur@PDA@GelCA exhibits excellent adhesive and antioxidant properties, while PDA@GelCA possesses antioxidant and anti-inflammatory properties, making it beneficial for treating diseases associated with oxidative stress and inflammation [83].

The effectiveness of scavenging free radicals and purifying the hydrogels was evaluated. The GelCA hydrogel demonstrated a free radical-scavenging efficiency of approximately 16.7%. It was found that increasing the concentration of nanoparticles enhanced both the ROS-scavenging efficiency and the purification effectiveness. At a nanoparticle concentration of 80 μg/100 μL of hydrogel, the ROS-scavenging efficiency improved to 72.1% and 91.9%, respectively. Additionally, these hydrogels were shown to protect retinal cells from oxidative stress-induced damage, with no evidence of tissue atrophy post-application, indicating excellent biocompatibility and non-toxicity [79,84].

The impressive properties of gelatin-based nanomaterials suggest their potential as a future solution for treating oxidative stress-related diseases such as dry eye syndrome.

#### 4.1.2. Hydrogels

Hydrogels are composed of polymer chains capable of binding large amounts of water. These polymers can be natural, semi-synthetic, or synthetic, including substances like methylcellulose, chitosan, and hyaluronic acid (HA). Their three-dimensional structure is key to their potential, as it facilitates the binding of various medicinal substances, enabling targeted drug delivery. Additionally, the physical properties of hydrogels can be adjusted by altering temperature, ionic strength, or pH, which controls the rate of degradation and supports prolonged drug release on the eye surface. Due to these properties, hydrogels are extensively utilized in ophthalmology as materials for eye drops, contact lenses, implants, eye patches, and platforms that carry reactive oxygen species-scavenging substances [85,86,87].

##### Soft Hydrogel Based on Hyaluronic Acid

A soft hydrogel based on hyaluronic acid was developed through the chemical cross-linking of a hyaluronic acid polymer modified with vinylsulfone (VS) and thiol (SH) groups. HA was chosen for its ubiquitous presence in the body and its versatile effects, as discussed earlier in the article. The study demonstrated that this hydrogel possesses high elasticity and viscosity similar to traditional eye drops, while also being comfortable to use without causing discomfort post-application [88,89].

The effectiveness of soft hydrogels in treating dry eye disease (DES) was evaluated in a study conducted on dogs. The hydrogel was administered using an eye-drop applicator twice daily, alongside ongoing cyclosporine treatment. The study found that dogs not responding to cyclosporine alone experienced reduced symptoms with the addition of the soft hydrogel. Improvements included increased tear production, reduced conjunctival hyperemia, decreased corneal inflammation, and overall enhancement in the structure of the eye surface. These benefits are attributed to the hydrogel’s high biocompatibility and its capability for prolonged drug release [89,90].

The soft hydrogel based on HA, due to its prolonged action, enables less frequent application without increasing the risk of aggravating the disease, thereby reducing treatment costs. An additional key benefit is the stabilization of the corneal tear film. Cross-linked hyaluronic acid, in comparison to its non-cross-linked counterpart, exhibits superior rheological properties and a viscosity that more closely resembles the natural condition of the eye surface. Considering all these factors, this formulation represents a promising advancement in the treatment of dry eye [90,91].

##### Thermosensitive Hydrogels

Thermosensitive hydrogels, such as those based on poly(N-isopropylacrylamide) (PNIPAM), are notable for their ability to alter physical properties with temperature changes. PNIPAM, which contains both hydrophilic and hydrophobic groups, transitions to a sol state below 32 °C, facilitating targeted drug release through minor temperature adjustments at the desired site. Despite its many benefits, PNIPAM-based hydrogels have notable drawbacks, including poor mechanical strength, limited drug-loading capacity, and low biodegradability. To overcome these limitations, it is suggested to blend PNIPAM with other polymers such as hyaluronic acid and carboxymethyl chitosan, which can enhance the mechanical properties and overall functionality of the hydrogel [92].

One of the applications of thermosensitive hydrogels is the targeted transport and release of drugs to hard-to-reach areas, such as the deeper layers of the eye. These hydrogels work by initially binding the drug; upon administration, they respond to temperature changes, altering their structure and state, which triggers the release of the encapsulated drug. Studies have shown that using thermosensitive hydrogels can achieve drug concentrations at the target site that are 100 times higher than those obtained through systemic drug administration. This delivery mechanism offers a less invasive alternative to traditional treatment methods, such as surgery. Ongoing research aims to enhance this technology, which already shows significant promise in treating conditions like dry eye syndrome [93,94,95].

##### Reactive Oxygen Species-Scavenging Hydrogels

Reactive oxygen species are a primary factor in the development of dry eye syndrome and other conditions related to oxidative stress [37,38]. In cases of dry eye syndrome, excessive ROS can cause irreversible damage to the retina and dysfunction of the meibomian glands, often exacerbating the disease and leading to persistent symptoms. Although the natural antioxidant defense system can reduce some of the excess ROS, it is often insufficient, prompting the search for new methods of ROS elimination [37,41,96].

ROS-scavenging hydrogels have become widely used in medical applications, aiding in wound healing, bone regeneration, and the treatment of eye diseases. These hydrogels provide antioxidant substances that effectively scavenge ROS, offering therapeutic benefits [96,97].

Nanoparticles encapsulated within hydrogel capsules can be either organic (e.g., phenol or aniline) or inorganic (e.g., cerium oxide, iron oxide, or manganese oxide). The action mechanism of these inorganic nanoparticles involves altering the redox potential by changing the oxidation state of the metals, which facilitates the removal of ROS from inflamed areas. By selecting different types of inorganic nanoparticles to incorporate into the hydrogels, it is possible to tailor the final therapeutic effects to specific needs [86,98,99].

Organic hydrogels, such as those containing phenolic groups derived from substances like dopamine, curcumin, or gallic acid, eliminate ROS through electron and proton transfer. Their potent antioxidant properties help protect cells from the damaging effects of ROS, thereby accelerating the regeneration of damaged tissues. Additionally, some of these hydrogels are enhanced with antibacterial agents, further aiding in the elimination of potential biological pathogens [86,100,101].

Hydrogels offer numerous advantages, including biocompatibility, flexibility, mechanical stability, and non-toxicity. By modifying their composition, it is possible to tailor their properties for optimal therapeutic effects. This versatility enables their widespread use across various medical and scientific research applications [86,102].

##### Lysine-Carbonized Mucoadhesive Nanogels

Lysine-carbonized nanogels (Lys-CNG), which are drug-free, carbonized nanomaterials, were studied for use in DES. These nanogels are produced from lysine hydrochloride through thermal polymerization and carbonization, transforming lysine into nitrogen-doped, cross-linked polymers. Pyrolysis of lysine hydrochloride was performed at temperatures of 240, 260, or 280 °C, resulting in Lys-CNG-240, Lys-CNG-260, and Lys-CNG-280, with Zeta potential values of 11.4, 22.8, and 37.3 mV, respectively. ZP analysis suggests that the cationic Lys-CNG-260 enhances mucosal adhesion and was found to be the most effective at reducing corneal epithelial damage in a rabbit model of DES [103].

Lysine influences the Nrf2 signaling pathway, which is dependent on the mitogen-activated protein kinase p38 (p38 MAPK/Nrf2). It increases Nrf2 levels and boosts the expression of antioxidant enzyme genes, thereby neutralizing oxidative stress [104,105].

Carbon nanomaterials, known for their free radical-scavenging activities, act as both electron donors and acceptors [106]. This dual functionality allows them to serve as either antioxidants or pro-oxidants. Their functional groups, namely carboxyl and amine, contribute to the antioxidation process by transferring a hydrogen atom, an electron, or both [107]. Some studies have shown that antioxidant activity is linked to the sp2 carbon network, which involves the formation of radical adducts, spin delocalization, and radical destruction [106]. Additionally, doping heteroatoms into nanomaterials is an effective strategy to enhance electron transfer, thereby improving nanozyme-mimicking activity [108].

Research indicates that Lysine-carbonized nanogels (Lys-CNG) possess antioxidant and anti-inflammatory properties, and their strong mucoadhesive properties increase retention time on the eye surface. This extended contact reduces the frequency of drug administration needed, demonstrating high biocompatibility with corneal epithelial cells both in vitro and in vivo. Lys-CNG is thus recognized as having significant potential as a long-term therapeutic agent for DES [103]. Moreover, carbon-based nanomaterials effectively remove ROS from mammalian cells and living tissues while reducing inflammatory responses with relatively few side effects, making them highly biocompatible and excellent candidates for nanodrugs in the treatment of eye diseases [109].

#### 4.1.3. Nanoemulsions

Nanoemulsions (NEs) are a stable, lipid-based form often used as artificial tears. Thanks to their properties that mimic the natural tear film, artificial tears help reduce tear evaporation from the eye surface, thus aiding in the regeneration of the damaged lipid layer [110]. NEs consist of water and oil in various combinations, allowing them to be either oil dispersed in water or water dispersed in oil. These formulations also include cationic surfactants. Due to their small size, nanoemulsions can easily penetrate barriers, facilitating rapid delivery of medications to the sites affected by the disease [111].

The water phase of the nanoemulsion hydrates the eye by enhancing the water layer of the tear film. The oil phase, once released, helps reduce tear evaporation by integrating with the lipid layer. Additionally, surfactants in the NE stabilize its structure and increase the bioavailability of the drug [112].

##### Cyclosporine A Nanoemulsion

Cyclosporine A is an anti-inflammatory drug used long-term to enhance tear production in the treatment of dry eye syndrome. CsA is traditionally available in an emulsion that includes castor oil and glycerin to improve its solubility [113]. However, due to adverse reactions such as burning and itching, adherence to medical recommendations for CsA emulsion has been challenging. Consequently, 0.05% CsA has been formulated into nanoemulsions that are non-toxic, non-irritating, and user-friendly. Studies have shown that CsA nanoemulsions offer improved stability, bioavailability, and treatment efficacy [114,115].

Studies have shown that 0.05% cyclosporine nanoemulsion, due to its small particle size, achieves better absorption than traditional cyclosporine emulsions. Consequently, the drug can penetrate deeper into the eye structures and remain effective for a longer duration. It was found that the use of CsA nanoemulsion reduces inflammation in the lacrimal gland, enhances the stability of the tear film, and increases the volume of tears produced. This increase is attributed to a rise in the number of goblet cells and enhanced mucin secretion [58]. Moreover, compared to CsA emulsion, nanocyclosporine demonstrated noticeable improvements in patient conditions as early as after four weeks of use, whereas traditional cyclosporine required up to twelve weeks to show similar effects. This accelerated effectiveness may be attributed to the reduced adverse reactions associated with nanocyclosporine, which facilitates more consistent and comfortable usage of the preparation [114,115].

##### Nanoemulsion of Propylene Glycol and Hydroxypropyl Guar (PG-HPG)

**Figure 8:**
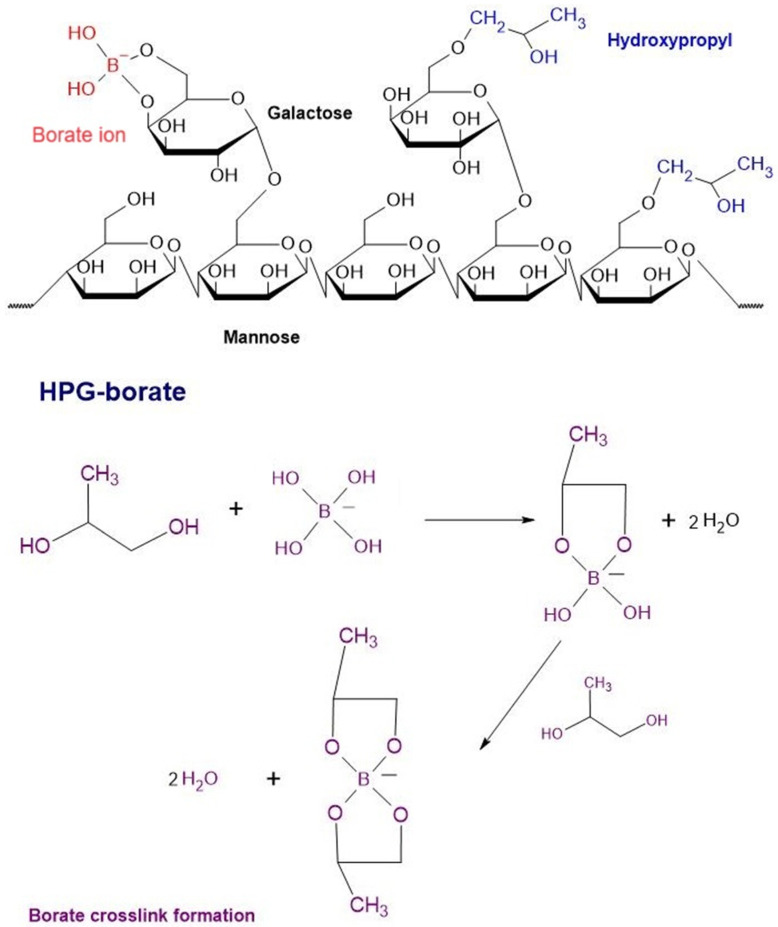
Chemical structures of HPG-borate and the formation of the borate cross-link.

Hydroxypropyl guar (HPG) serves as a viscosity-increasing and thickening agent in eye drops. When combined with borate, it enhances the retention of active soothing agents like propylene glycol. Additionally, antibacterial agents are incorporated to mitigate the risk of contamination during production, and sorbitol is used to optimize the viscosity of the drops. These drops are specifically designed for individuals with DES caused by lipid or water deficiencies (Figure 8) [116].

The PG-HPG nanoemulsion works by forming a protective barrier on the surface epithelium through an HPG/borate mesh. This mesh facilitates the secretion of lipids into the tear film, maintaining its function even when the eye’s pH is stabilized [116].

The anionic phospholipid DMPG (dimyristoyl phosphatidylglycerol) is released to fill the lipid gaps caused by dry eye syndrome. This action helps reconstruct the eye’s structure, reducing friction between blinks and maintaining the stability of the tear film. By ensuring an adequate lipid level, it also reduces inflammation triggered by oxidative stress, thus preventing disease progression [117]. Furthermore, studies indicate that PG-HGP nanoemulsion is also beneficial for contact lens wearers, extending the duration of comfortable lens use by alleviating eye dryness [118].

The release of the anionic phospholipid DMPG in PG-HPG nanoemulsion eye drops has been shown to effectively treat patients with DES, without the risk of adverse reactions. Many symptoms of DES are alleviated after just 28 days of use, allowing for the restoration of ocular surface health and controlling the progression of the disease [119,120].

#### 4.1.4. Nanopreparation: Polyphosphobetaine Functionalized with p(MPC-Co-DMA) Catechols

A catechol-functionalized polyphosphobetaine copolymer, p(MPC-co-DMA), composed of 2-methacryloxyethylphosphorylcholine (MPC) and dopamine methacrylamide (DMA) monomers, was synthesized through random free radical copolymerization [121]. In this copolymer, DMA forms a hydrophobic core while MPC contributes hydrophilic coatings [122]. The polymer, p(MPC), is zwitterionic, containing equal numbers of cationic and anionic functional groups. This structure allows water molecules to form a permanent hydration layer around the polymer, attracted by ionic solvation to the charged functional groups [123]. Due to its hydrophilic nature, p(MPC) effectively retains water on the cornea, thereby reducing dehydration of the eye [124].

P(MPC) is engineered to mimic the structure of human biomembranes, which minimizes adverse bodily reactions [125]. The DMA monomer plays a key role in scavenging reactive oxygen species, thereby extending the retention time on the ocular surface and prolonging the copolymer’s therapeutic effects [121]. The ROS-scavenging ability of p(DMA-co-MPC) is largely due to the hydroquinone groups present in the DMA components. Studies on ROS removal in vitro using p(DMA-co-MPC) demonstrated significant efficacy: at a 4.0 mg/mL concentration in a 1:1 molar ratio, more than 95% of O2•– radicals were eliminated, and at 2.0 mg/mL, 83% removal efficiency was achieved for O2•–. When the concentration was increased to 4.0 mg/mL, nearly all OH• radicals were removed. This indicates that the antioxidant capacity of p(DMA-co-MPC) is directly proportional to its DMA content [122].

**Figure 9:**
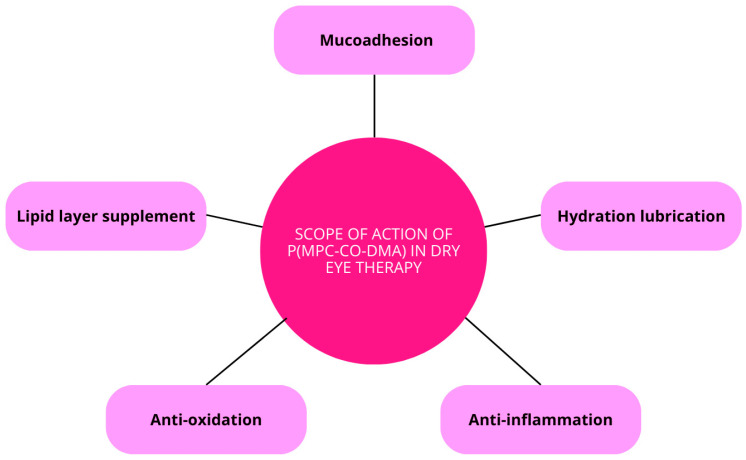
Scope of action of p(MPC-co-DMA) in dry eye therapy.

The combination of MPC’s lubricating effects and DMA’s radical-scavenging ability enables the nanoagent to effectively alleviate both tear-film hyperosmolarity and corneal inflammation. Research indicates that after a single application of p(MPC 1-co-DMA 1) at a 1 mg/mL dosage, significant inhibition of cell apoptosis and the expression of pro-inflammatory factors such as IL-6 and TNF-α can be observed within four days. These properties underscore the potential of this nanoformulation as a promising bioactive eye drop for DES treatment (Figure 9) [121].

#### 4.1.5. Carbohydrate-Based Nanomaterials (Charged Polysaccharides—Glycosaminoglycans)

Carbohydrates are naturally occurring biomolecules involved in numerous metabolic processes, including enzyme transport, cell migration, immune responses, and intercellular interactions. Their structural diversity and unique properties such as low toxicity, biodegradability, and hydrophilicity have made them ideal for synthesizing nanocarriers [126].

These nanomaterials, which can be derived from complex polymers or monosaccharides, gain enhanced utility through chemical modifications. Additionally, the abundant hydroxyl groups in polysaccharides facilitate non-covalent bioadhesion with tissues, which improves drug targeting. Consequently, carbohydrate-based nanomaterials are utilized in various fields including tissue engineering, modern drug delivery systems, and energy, with promising potential for biomedical applications [127].

##### Hyaluronic Acid

**Figure 10:**
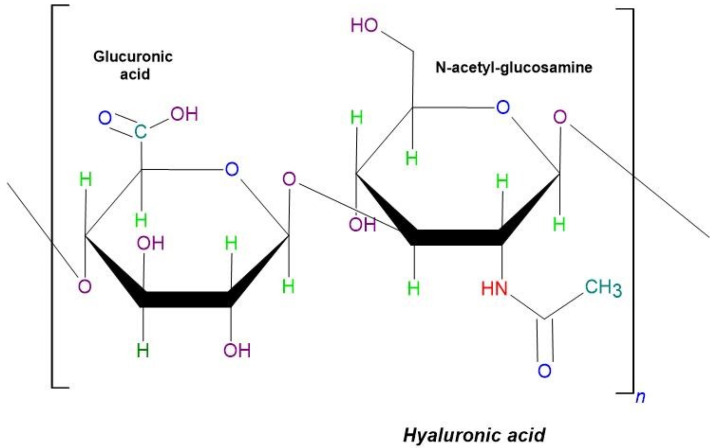
The schematic structure of hyaluronic acid.

Hyaluronic acid is a large, linear polysaccharide composed of repeating units of N-acetyl-d-glucosamine and d-glucuronic acid, which are linked by β-1,3 or β-1,4 glycosidic bonds (Figure 10). It is synthesized by most cells at various stages of the cell cycle, making it ubiquitous throughout the body. HA plays critical roles in numerous biological functions, including cell proliferation, differentiation, tissue regeneration, and remodeling processes [128].

HA is found in high concentrations in soft connective tissues and is also naturally present in the tear film, outer cornea, and vitreous humor. Its properties and biological effects can be tailored by altering its molecular weight or concentration, was well as through chemical modifications. For instance, low molecular weight HA exhibits pro-inflammatory properties and lower viscosity, whereas high molecular weight HA is characterized by anti-inflammatory properties and greater viscosity [129]. Given its anti-aging, anti-proliferative, immunomodulating, and tissue-repairing properties, HA is increasingly viewed as pivotal to the future of the biomedical industry, including ophthalmology [130,131].

Hyperosmolarity of the tear film is a primary factor in the development of dry eye syndrome, leading to ocular surface damage, inflammation, and pain [132]. HA, with its high content of hydroxyl groups that attract water molecules, significantly enhances the properties of the tear film. Literature reviews indicate that treating DES with hyaluronic acid increases the viscosity of the tear film, prolongs its retention on the eye surface, ensures its even distribution, and reduces the risk of mechanical trauma. It also positively affects the health of the meibomian glands [130,132].

The ability to modify the physicochemical properties of HA allows for customization of eye-drop formulations to achieve optimal therapeutic effects [130]. This is supported by a study on the complex formed between HA nanoparticles and MitoQ nanoparticles. The research focused on the impact of temperature on the formation of the HA/MitoQ nanoparticle complex and its subsequent biological effects. Using elevated temperatures increased the interactions between the nanoparticles, enhancing the antioxidant activity of the complex. Moreover, when compared to free MitoQ, the HA/MitoQ complex demonstrated superior therapeutic effects in treating dry eye syndrome (DES). Specifically, it reduced the accumulation of ROS and alleviated DES symptoms more effectively [133].

Another notable example is the use of gelatin gallate and epigallocatechin nanoparticles decorated with hyaluronic acid (GEH) as eye drops. Studies on their in vitro biocompatibility and therapeutic effects on the corneal epithelium were conducted. It was observed that GEH primarily accumulated in the cytoplasm of the corneal epithelium and on the eye surface, demonstrating its effectiveness in drug delivery to the eye. Furthermore, GEH not only avoided causing undesirable changes but also effectively reduced the symptoms of dry eye syndrome [134].

These examples underscore the therapeutic potential of HA, showcasing its healing capabilities and the beneficial effects it can produce. Moreover, the positive impact of HA nanoparticles on the well-being of patients suffering from dry eye syndrome has been confirmed. The accumulating evidence supports the promising role of HA nanoparticles in both nanomedicine and the targeted therapy of DES [133,134].

#### 4.1.6. Metallic and Non-Metallic Nanomaterials

Due to the blood–retina barrier, which regulates the transport of molecules through the eye, and natural phenomena such as tearing or blinking, traditional treatment methods often fail to deliver satisfactory therapeutic effects. Attempts have been made to use intraocular injections to bypass this barrier, but the approach has not been widely adopted due to numerous side effects [10].

In response to these challenges, nanoparticles have gained significant attention in recent years. Their small size, specific shapes, and stability make them particularly promising for medical applications, offering potential new pathways for the treatment of eye diseases [11].

Various methods are employed to synthesize them, including chemical reduction, synthesis reaction, electrochemical, and sonochemical techniques. By tweaking these synthesis methods, it is possible to tailor the properties of nanoparticles to enhance their biological effectiveness. Factors such as chemical charge, size, shape, and solubility can be adjusted [10,11].

Types of nanoparticles include metal oxide nanoparticles, metal nanoparticles, doped nanoparticles, and metal-organic frameworks. Some metal nanoparticles can have undesirable effects, such as stimulating inflammatory processes through excessive ROS production. However, not all metal nanoparticles behave detrimentally; some, like gold and cerium oxide nanoparticles, are known for their therapeutic effects [135].

##### Selenium and Copper

Selenium is a trace element essential for numerous physiological processes in the body, including antioxidant defense, thyroid hormone production, and redox homeostasis. It exists in various forms such as selenocysteine, selenium-methyl-selenocysteine, selenomethionine, selenate, and selenite, each with distinct bioavailability and properties. However, it is crucial to maintain selenium concentrations within the reference range to prevent cytotoxic effects. Therefore, strict monitoring of selenium levels is essential to ensure its beneficial effects without risking toxicity [136].

Selenium nanoparticles (SeNPs) have gained attention for their selective toxicity toward cancer cells, while sparing the body’s normal physiological cells. Extensive research has highlighted the potential therapeutic effects of SeNPs in various fields, including oncology, neurology, and metabolic disorders. These nanoparticles have also demonstrated the ability to interact with a wide range of chemical compounds, leveraging their adsorption capacity for broader medical applications. This versatility extends to ophthalmology, where SeNPs may offer new treatment possibilities [137].

Copper is an essential element that plays a crucial role in numerous enzymes, including superoxide dismutase, tyrosinase, and lysine oxidase [138]. Copper-based nanomaterials exhibit higher antimicrobial activities compared to silver-based nanoparticles, making them particularly effective in medical applications. Additionally, the distinctive porous structure of copper nanoparticles facilitates surface modifications with other compounds, significantly broadening their potential uses in medicine [139].

This thesis is supported by an experiment involving F127 hydrogel containing copper selenide nanoparticles, used in a new formulation of eye drops for treating DES [138]. The study by Ou et al. [138] showed that selenium nanoparticles have strong antioxidant, anti-inflammatory and anti-apoptotic properties. These nanoparticles were specifically effective in scavenging reactive oxygen species, thereby reducing oxidative stress, which is a primary contributor to the development of DES. Consequently, the treatment significantly slowed the apoptosis of corneal and retinal cells.

The study utilized a kit where molybdenic acid forms a complex with hydrogen peroxide. This setup allowed for the precise measurement of the rate at which copper selenide nanoparticles capture hydrogen peroxide. The primary mechanism of action for Cu2-xSeNP involves modulating the p38MAPK and NRF2 signaling pathways. These findings suggest that these nanoparticles could be effective in treating dry eye syndrome, although additional research is needed to fully assess their suitability for this application [138].

##### Cerium Oxide

Cerium oxide nanoparticles, or nanoceria, are among the most commonly discussed nanozymes in medical literature [140]. A nanozyme is a type of nanomaterial designed to mimic the action of enzymes, which are often implicated in the development of various disorders. Nanoceria’s unique feature is its dual valence states of +3 and +4, which facilitate electron movement. This property underscores its capability to effectively scavenge reactive oxygen species [140,141].

Studies have demonstrated that nanoceria possess anti-inflammatory, anti-apoptotic, and antioxidant properties and are effective in protecting against retinal dysfunction. Common formulations include nanoceria coated with glycolic chitosan, embedded in hydrogels, or incorporated into contact lenses [142].

The small size of cerium oxide nanoparticles enables them to penetrate deeper layers of the eye, enhancing defense mechanisms and aiding in the repair of damaged eye structures. This capability highlights their potential use in treating diseases associated with oxidative stress, including various ophthalmological conditions [142].

Glycol chitosan (GC) is a water-soluble, non-toxic, and biocompatible derivative of chitosan. When combined with nanoceria to create glycol chitosan cerium nanoparticles (GCCNPs), the resulting composite exhibits enhanced antioxidant and healing properties, making it especially beneficial for treating dry eye syndrome. GCCNP offers greater solubility than cerium oxide alone and is free from side effects [141].

Current treatments for DES focus on alleviating symptoms, restoring the normal tear-film composition, and reducing inflammation. Leveraging the properties of GC and nanoceria, researchers have dissolved cerium in GC to assess its impact on the ocular structure affected by DES. Experimental results show that GCCNP stabilizes the tear film, stimulates cell proliferation, provides antioxidant effects, and preserves the normal structure of the eye [141].

Cerium oxide nanocrystals attached to stem cell exosomes represent a novel therapeutic strategy for diseases linked to excessive ROS activity. Eye drops formulated with these nanocrystals on exosomes (MSCExo-Ce) leverage the regenerative, anti-inflammatory, and ROS-scavenging properties of both components. Research has shown that MSCExo-Ce effectively scavenges ROS from affected areas, reducing inflammation and promoting tissue repair. These promising results lay the groundwork for further studies in developing advanced therapies for the treatment of DES [143].

## 5. Therapeutic Contact Lenses to Remove Excess Reactive Oxygen Species from the Surface of the Eye

Therapeutic contact lenses encapsulated with polymer nanoparticles are produced through a two-step process: first, the drug-containing nanoparticles are synthesized, and then these nanoparticles are embedded into the contact lens matrix [144].

Specifically, water-soluble contact lenses incorporating cerium nanoparticles (CeNP-CLs) was developed using polyhydroxyethyl methacrylate (PHEMA) as the matrix. Additionally, 1-vinyl-2-pyrrolidinone (NVP) and methacrylic acid (MAA), a negatively charged compound, were included. The electrostatic interaction between the negatively charged MAA and the positively charged CeNPs enhances the stability of the nanoparticles within the lens, preventing unwanted release. Notably, CeNPs are known to mimic the enzymatic activities of catalase and superoxide dismutase, contributing to their therapeutic effects [96].

Cerium-based materials due to the presence of dual valence states Ce^3+^ and Ce^4+^ have the ability to reduce ROS [145].

Research has demonstrated that increasing the concentration of cerium nanoparticles (CeNP) in cerium nanoparticle-loaded contact lenses (CeNP-CLs) correlates directly with the rate of ROS removal from the ocular surface. While CeNP-CLs are effective in reducing extracellular ROS, they do not impact intracellular ROS. Nevertheless, they can significantly reduce damage to the corneal epithelium and endothelium. Furthermore, CeNP-CLs display properties—including transparency, dimensions, and elasticity—comparable to commercially available contact lenses, making them promising candidates for the treatment of dry eye syndrome [96].

## 6. Conclusions

Dry eye syndrome is one of the most prevalent eye conditions, yet current treatments often fall short due to rapid drug clearance from the ocular surface and low bioavailability. As a result, there is a growing interest in alternative drug delivery methods. Nanomaterials, in particular, show promise as drug delivery systems, offering enhanced retention on the ocular surface and improved targeting of the anterior segment of the eye.

Extensive research is underway to optimize nanocarriers for increased effectiveness, focusing on extending their contact time, improving bioavailability, and ensuring precise drug delivery. Notably, some polymer nanomaterials have already received FDA approval, while others are currently undergoing clinical trials, underscoring their potential and the expanding focus on their development.

## Figures and Tables

**Table 1:** The examples of mechanisms of action of internal factors on the development of dry eye syndrome.

Etiology	How It Leads to DES	References
Hormonal imbalance	Androgens have been demonstrated to regulate the lacrimal glands’ fluid and protein secretion by steroid-specific receptors in epithelial cells. The effect of the lack of androgens is dysfunction of the lacrimal glands and deficiency of tears.	[15,16,17,18]
Gut dysbiosis	Gut dysbiosis stimulates the migration of CD103 or CXCR1 dendritic cells, or monocytes/macrophages, to the surface of the eye. This contributes to the activation of T lymphocytes to secrete pro-inflammatory cytokines on the surface of the eye and lacrimal glands.	[19,20]
Autoimmunity	Activated CD8 T cells are associated with the death of lacrimal gland epithelial cells, reducing tear production. CD4 T cells, as the main immune effectors, interact with macrophages, causing inflammation and peripheral neuropathy of the lacrimal glands.	[21,22]
Nerve damage	Nerve damage causes a decrease in the sensitivity threshold of sensory neurons or their excessive excitation. This is related to malfunctioning ion channels affecting the generation and propagation of action potentials.	[23,24]

**Table 2:** The structure of the tear film [5,28,33,34,35,36].

Type of Layer	Composition	Function
Lipid layer (outer layer)	Non-polar wax esters (25.2%, e.g., fatty esters, fatty alcohols), phospholipids (4.5%), fatty acids (3.5%), and cholesterol (free and esters 66.8%)	Delaying the evaporation of tears Uniform distribution of the tear film Maintaining a smooth eye surface
Aqueous layer (middle layer)	Proteins (lysozyme, lactoferrin), metabolites (includes peptides, lipids, amino acids, nucleic acids, carbohydrates, vitamins), inorganic salts (NaCl), glucose, oxygen, and electrolytes (magnesium, bicarbonate, calcium, urea)	Flushing out impurities and toxins Moisturizing and protecting the surface of the eye Calcium ions are essential for cell adhesion, aiding in the stabilization and integrity of the ocular surface by facilitating cell-to-cell and cell-to-matrix interactions. Magnesium ions, meanwhile, act as coenzymes in various protective processes on the eye surface, including stabilization of cellular membranes and modulation of oxidative stress responses.
Mucin layer (inner layer)	Immunoglobulins, urea, inorganic salts, glucose, and proteins	Ensuring even lubrication of the eye Lowering surface tension and increasing the stability of the tear film

**Table 3:** The classification of oxidative stress according to intensity.

Intensity Classification	Characteristics
Basal oxidative stress	Very low intensity of oxidative stress
	No apparent symptoms Oxidative–redox homeostasis
Low-intensity oxidative stress	Slight increase in the level of ROS modifiable molecules
	Increased activity of antioxidant enzymes
Strong oxidative stress	Disturbed balance between oxidants and antioxidants
	Significant predominance of oxidants
Very strong oxidative stress	Maximum level of modifiable ROS particles
	Minimal activity of antioxidant enzymes

**Table 4:** The classification of oxidative stress according to time course.

**Time-Course Classification**	**Characteristics**
Acute oxidative stress	Short-term increase in ROS levels, with change of conditions (e.g., use of an oxidative stress inducer)
	Highly effective antioxidant defense, restoring homeostasis Presumed lack of stimulation of the expression of ROS-neutralizing genes
Chronic oxidative stress	Long-term elevated levels of ROS, with change in conditions (e.g., use of an oxidative stress inducer)
	Strong stimulation of the expression of ROS-neutralizing genes, due to weakened antioxidant defence

## Data Availability

Available on request and with regulations.
